# Bibliographical

**Published:** 1872-01

**Authors:** 


### BIBLIOGRAPHICAL.

*General Surgical Pathology and Therapeutics.* In Fifty Lectures. A Text-Book for Students and Physicians. By Dr. Theodore Billroth, Professor of Surgery in Vienna. Translated from the Fourth German Edition, with Special Permission of the Author, by Charles E. Hackley, A.M., M.D., Surgeon to the New York Eye and Ear Infirmary; Physician to the New York Hospital; Fellow of the New York Academy of Medicine, etc., etc. New York: D. Appleton & Company.

We regret that our limited space will not permit us to do more than merely glance at some of the most prominent features of this most excellent work. The fact, no longer contested, that German pathologists at present stand preëminently in advance of all others, and that Professor Billroth is one of the most noted authorities on surgical pathology, at once gives that importance to the work, as authority, which few possess. Surgical pathology, as the title suggests, is one of the most prominent features of this work, and more particularly the recent advances in this department of the sciences, but so interspersed with the description of disease and practical therapeutical suggestions as to relieve it of that dryness so common to works on pathology.

He has effectually cut loose from the old routine of the text-book, and, by so doing, has exploded many of the traditions which have been handed down from one text-book to another. It is comprehensive and concise, and characterized by great clearness and originality. It is not only a book for the student, but a work of reference to the physician. The fact that it has passed through four editions in a short time, and been translated into French, Italian, Russian, Hungarian and English, is some evidence of the value of the work. We regard it as one of the best works upon the subjects treated that we have seen.

We have received a number of other books, etc., which will be noticed in the next number of the JOURNAL.

